# P02.57. Mindfulness meditation versus exercise in the prevention of acute respiratory infection, possible mechanisms of action: a randomized controlled trial

**DOI:** 10.1186/1472-6882-12-S1-P113

**Published:** 2012-06-12

**Authors:** A Zgierska, C Obasi, R Brown, T Ewers, D Rabago, B Barrett

**Affiliations:** 1 University of Wisconsin, Department of Family Medicine, Madison, USA

## Purpose

The original randomized controlled trial (RCT) compared mindfulness meditation and exercise to a control condition and showed that these interventions may be effective in reducing acute respiratory infection (ARI) illness burden and costs. Differential effects of these interventions were noted. The goal of this analysis is to evaluate possible mechanisms underlying efficacy.

## Methods

The study design was a 3-arm non-blinded RCT. Participants were community-recruited adults ≥ 50 years old. Group 1 received meditation training; Group 2 “matching” moderate-intensity exercise training; and Group 3 were a waitlist control. Outcome Measures were assessed at baseline, 9 weeks (post-intervention) and 3 months. Primary outcomes were ARI duration and severity (area-under-the-curve global severity, Wisconsin Upper Respiratory Symptom Survey). Secondary outcomes were psycho-physical health questionnaires. Exercise minutes were tracked.

## Results

Of 154 randomized, 149 completed the trial and were included into analysis. Participants were on average 59 (standard deviation 6.6) years old; 82% were women, 94% were Caucasian and 93% were non-smokers. Two age/gender-adjusted hypothesis-driven mediational models assessed effects of selected variables on primary outcomes. Stage 1 model contained group status and the change in a selected variable score over time; Stage 2 model additionally adjusted for the change in mindfulness scores. Stage 1 modeling showed that change in mindfulness scores at 3 months mediated the treatment effects on cold duration (p<0.05). Stage 2 modeling (double mediation structure) showed that changes in Optimism, Social Support and Mental Health scores at 3 months influenced (p<0.05) cold duration; however, these effects appeared to be mediated (p<0.05, p<0.05, and p=0.056, respectively) by the change in mindfulness scores at 3 months, with larger increases in mindfulness scores correlating with a shorter ARI duration. Change in exercise minutes did not appear to have significant effects on primary outcomes.

## Conclusion

Positive effects of meditation and exercise on ARI illness burden may be explained by improved mindfulness scores over time. These results call for further research.

